# Technologies for Assessment of Motor Disorders in Parkinson’s Disease: A Review

**DOI:** 10.3390/s150921710

**Published:** 2015-08-31

**Authors:** Qi Wei Oung, Hariharan Muthusamy, Hoi Leong Lee, Shafriza Nisha Basah, Sazali Yaacob, Mohamed Sarillee, Chia Hau Lee

**Affiliations:** 1 School of Mechatronic Engineering, Universiti Malaysia Perlis (UniMAP), Campus Pauh Putra, 02600 Arau, Perlis, Malaysia; E-Mails: hari@unimap.edu.my (H.M); hoileong89@gmail.com (H.L.L.); shafriza@unimap.edu.my (S.N.B.); sarilleelee@gmail.com (M.S.); mrlionryan@gmail.com (C.H.L.); 2 Universiti Kuala Lumpur Malaysian Spanish Institute, Kulim Hi-TechPark, 09000 Kulim, Kedah, Malaysia; E-Mail: sazali.yaacob@unikl.edu.my

**Keywords:** Parkinson Disease (PD), wearable sensor, audio sensor, multimodal sensor, Unified Parkinson Disease Rating Scale (UPDRS)

## Abstract

Parkinson’s Disease (PD) is characterized as the commonest neurodegenerative illness that gradually degenerates the central nervous system. The goal of this review is to come out with a summary of the recent progress of numerous forms of sensors and systems that are related to diagnosis of PD in the past decades. The paper reviews the substantial researches on the application of technological tools (objective techniques) in the PD field applying different types of sensors proposed by previous researchers. In addition, this also includes the use of clinical tools (subjective techniques) for PD assessments, for instance, patient self-reports, patient diaries and the international gold standard reference scale, Unified Parkinson Disease Rating Scale (UPDRS). Comparative studies and critical descriptions of these approaches have been highlighted in this paper, giving an insight on the current state of the art. It is followed by explaining the merits of the multiple sensor fusion platform compared to single sensor platform for better monitoring progression of PD, and ends with thoughts about the future direction towards the need of multimodal sensor integration platform for the assessment of PD.

## 1. Introduction

For the past several decades, quantitative monitoring of human motor control and movement disorders has been an evolving research field, which grown through the large global computer technologies, context-aware computing, solid-state micro sensors and telecommunication. This effective research has a continuous number of useful fundamental applications, with much attention-grabbing advances in term of human behavior modelling, interaction between human and machine, and healthcare field of research. In principle, this indeed will convey great public benefits, particularly in the applications related to human real life, for instance, attention towards healthcare technologies and elderly care. Beginning from the 1960s, the accessibility of advanced equipment has permitted many hospitals to measure human motor performance in details with good precision. This advanced equipment has also been used for studying various pathologies of human motor performance [1,2,3,4,5]. Research studies state the fact that Malaysia has been undergoing progress in term of health, extended life expectancy, lower mortality rate and decreasing of the fertility rate. This had taken about changes in the demographic profile of its population, where one of the main medical concerns it brings is the growth in the number of people affected by numerous types of illness as this prevalence increases exponentially with advancing age [6]. For a population that is shifting towards an older age range, Parkinson disease (PD) is categorized in second ranking for the commonest chronic progressive neurodegenerative disorder in the world after Alzheimer’s disease [7], which affects approximately 3% of people above 65 years old. For the coming 30 years, this figure is expected to double due to the increase in the number of elderly people, as age is the main key risk feature for the start of PD [8,9]. 

According to the World Health Organization (WHO), it was estimated that the world is having seven to 10 million PD patients. The incidence of Parkinson’s increases with age and the syndrome rates rise sharply after 60 years old. PD has greater impacts in North America and European countries compared to Africa or Asian countries, and men are 1.5 times more likely to have Parkinson’s compared to women [10,11]. In Malaysia, the Malaysian Parkinson’s Disease Association estimated that about 15,000 to 20,000 patients suffer from PD, where this figure is estimated to rise for the forthcoming centuries [10]. The most general symptoms of PD are tremor (uncontrolled trembling or shaking movements), bradykinesia (slowness of movement), akinesia (loss of control in producing motion), hypokinesia (decreased in body movement), rigidity (struggle to externally carry out movements), postural instability and falls, speech and swallowing difficulties. It is also linked with some other non-motor symptoms that consist of fatigue, nervousness, gloominess, slow thinking, difficult to focus, visual hallucinations, pain, urinary regularity or urgency, extreme sweating, and sleep deprivation (e.g., dream-enacting behavior with shouting or kicking during sleep, or excessive sleepiness during the day) [12,13,14,15,16]. PD is recognized as one kind of neurodegenerative disorder of the central nervous system that is categorized into the group of circumstances known as motor system disorders, which are due to the loss of dopamine-producing brain cells. Till now, identifying the reason that causes PD is still remain elusive and there is no existing treatment, though medication through drugs can relieve some of the symptoms in PD. Current therapy in managing PD symptom severity is through the replacement of dopaminergic agonist, via levodopa, combined with carbidopa, a peripheral decarboxylase inhibitor (PDI) that provides the greatest anti-Parkinsonian benefits to patients with Parkinson (PWP) [17,18,19,20]. 

**Figure 1:**
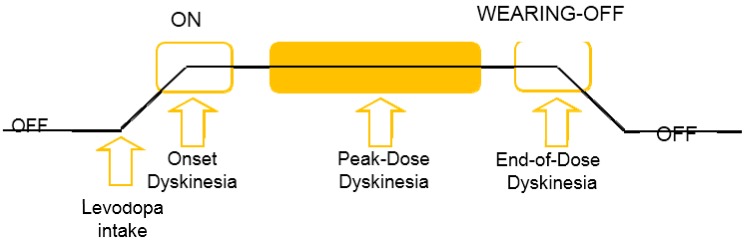
Schematic diagram illustrating the motor fluctuations cycle of PD.

Usually, levodopa, which has been the most successful medication in reducing Parkinsonian symptoms, is prescribed to these patients for eliminating the typical symptoms of PD. This therapy is effective during the initial stages of PD. Yet, in the PD’s later stage, PWP have developed motor difficulties that include sudden loss of efficiency of the medicine during the end of each treatment break, wearing off and uncontrolled hyperkinetic actions denoted as dyskinesia [9,17,19]. These variations are referred as motor fluctuations by the clinicians as shown in Figure 1. Many PWP start to fluctuate between the “off” state (*i.e*., re-emergence of PD symptoms due to the effect of levodopa wears off a few hours after levodopa intake) and the “on” state (*i.e*., levodopa is active and improves the patients’ motor performance). While, in the “on” state, patients had chances to suffer from dyskinesia. The presence of dyskinesia is a side effect of levodopa therapy and therefore denoted as levodopa-induced dyskinesia (LID) [21,22].

**Figure 2:**
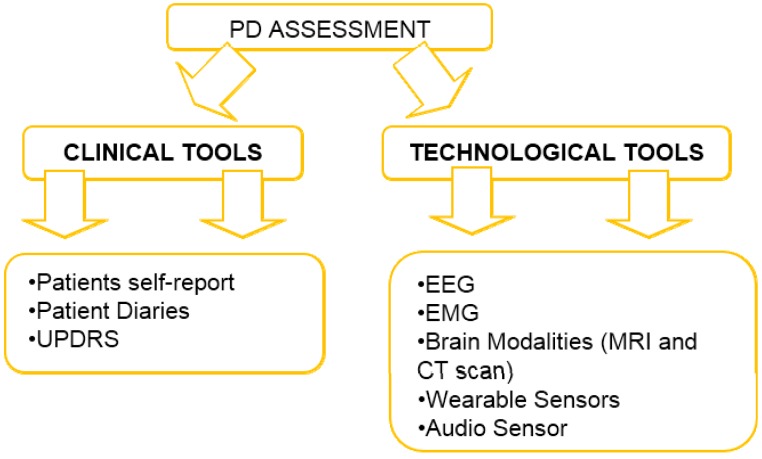
Summary of overall assessment of PD.

To ensure that these patients are able to be self-independent, clinician’s in-charge must have a precise picture of how the PWP symptoms will fluctuate throughout their everyday activities by optimally adjusting the medications. With the latest advancement in healthcare technology, techniques for PD symptom severity detection and assessment are pretty restricted. The validation of PD can be accomplished either through subjective clinical assessments or through objective technological tools. Figure 2 shows the summary of various types of assessments that are applicable in monitoring PWP.

### Assessment of Parkinson Disease-State of Art

One of the currently available tools for monitoring motor fluctuations of PWP is through subjective clinical practice. From the clinical side of view, patient-diaries, patient-self reports and prolonged observations on the spot approach have been applied. Details about motor fluctuations of PWP are obtained by using self-reports or the use of patient diaries. In order to obtain information regarding the motor fluctuations, PWP are requested to refresh back the total periods of active time and non-active time they had undergone. “Active time” is referring to the duration when the medicine is still active in weakening the indications of PD while “Non-active time” is referring to the duration of presence of PD symptoms.

However, these both solutions have drawbacks of recalling bias, for instance, patients frequently have trouble in differentiating dyskinesia from other types of symptoms. Even though the use of patient diaries can increase the reliability through the records as the symptoms occur, but this method only provides little information and does not collect useful features, which are advantageous for the clinicians to make an accurate judgment. Besides that, PD expert’s observations on the spot are unrealistic as the duration of motor fluctuations are more than a few hours between the medication prescriptions. The current existing conventional methods have many limitations, for instance, the requirement of patient’s frequent visits to the clinic that may be very inconvenient for them [9,19,23,24]. 

**Figure 3:**
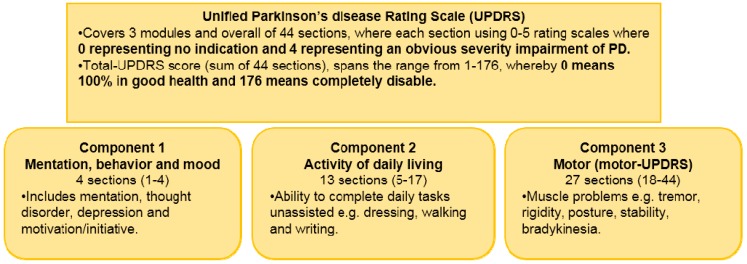
Overview of the clinical metric-Unified Parkinson Disease Rating Scale UPDRS (adapted from [26,27]).

In order to overcome these difficulties and looking for more objective assessment, numerous types of rating scales have been taken into account and applied. This method for PD symptoms monitoring typically requires expert clinical staff to conduct several practical tests and physical examinations. This is linked to the international gold standard reference scale, Unified Parkinson Disease Rating Scale (UPDRS) used by physicians, which reflects the presence and severity of PD symptoms. Figure 3 shows the summary of the clinical rating scale, UPDRS that computes the average PD symptoms severity. Unfortunately, the use of UPDRS brings some boundaries such as intra and inter observer inconsistencies whereby this scale may be too time consuming to administer and it can hardly be applied for continuous registration procedures done in the clinic. Additionally, UPDRS only offers assessment at that particular moment, but the symptoms severity of PWP may fluctuate extensively over the whole day. The motor fluctuation measurements taken during visits to the clinic might not precisely reveal the real functional disability experienced by patients while they are at home [25,26,27]. Prolong period of hospitalization will cause problems for the patient and their family members in term of financial status. Currently, this issue is one of the most demanding difficulties faced with PD as the appropriate medical care is progressively difficult and expensive. 

With the existing and on-going advance development in microelectronics, it had increased interest in using computerized methods for detecting early symptoms of PD on a more objective basis. This can be categorized into five groups: (1) techniques that analyzed electromyography (EMG) signals; (2) techniques that analyzed electroencephalogram (EEG) signals; (3) techniques based on 3-D motion analysis or imaging modalities (Computed Tomography (CT) scans or Magnetic Resonance Imaging (MRI)); (4) techniques examining motion signals using unimodal wearable sensors; and (5) techniques using audio sensors. The researches using such sensors for monitoring and detecting early symptoms of PD allow the opportunity to visualize an unremarkable system on a more continuous basis. These objective assessments are favorable tools that allow long-term home-based intensive care, having the possibility of improving the standards, delivering healthcare and at the same time, turning it into an effective and cost-saving procedure in PD progression. In the previous year, many advances have been conducted, but there is still an absence of an all-comprehensive system that had the capability in dealing with consistent PWP status assessment and at the same time economically reasonable. In this review, our focus will be on the state of art in early detection of PD symptoms severity performing through technological tools. The main objective of this review is to deliver a discussion of the abilities of different types of assessments of PD through technological tools, which are presented in the following section.

## 2. Related Research on PD advancement through Technological Tools

### 2.1. Monitoring on Progression of PD Using Electromyography (EMG)

The approach formerly conducted by previous researchers regarding EMG signals for differentiating PWP from healthy controls can be allocated into four categories: (1) non-linear analysis techniques [28,29]; (2) morphology analysis techniques [30,31]; (3) spectral based analysis techniques [28,32,33]; and (4) techniques from the above three groups. Overall summary of prior researches done on monitoring and detecting PD using EMG signals are shown in Table 1. 

**Table 1:** Summary of previous works conducted using EMG signals.

First Author and Year	Database	Techniques	Best Performance Measure
**Gennaro de Michele (2003) [32]**	16 male subjects (10 PWD and 6 healthy controls)	Wavelet correlation analysis with Global wavelet power (PCQ) parameters extracted from local wavelet power spectra	Accurately classify the PWP from healthy controls
**Saara Rissanen (2007) [30]**	48 subjects (26 PWP and 22 healthy controls)	Histogram and crossing rate (CR) values applied as high dimensional feature vectors and the dimensionality was reduced using Korhunen-Loeve transform (KLT)	Precise discrimination for healthy controls: 86% and PWP: 72%
**Saara Rissanen (2008) [34]**	33 healthy young controls 26 healthy old controls and 42 PWP	1. Selected features (six from right side and six from left side variables): (1). Kurtosis variable (2). CR variable 3. Correlation dimension 4. Recurrence rate 5. Sample entropy 6. Coherence variable	Clustering analysis using k-means algorithms into 3 clusters: One cluster having 90% of the healthy controls while the two other clusters having 76% of PWP
**A.I.Meigal (2008) [29]**	−19 PWP (4 men and 15 women), −20 healthy old controls (7 men and 13 women) −20 young controls (10 men, 10 women)	Non-linear SEMG features (% Recurrence, % Determinism and SEMG distribution kurtosis, correlation dimension and sample) entropy)	Differentiate PWP from healthy controls
**Bryan T.Cole (2010) [35]**	4 PWP and 2 healthy controls	1. Linear classifier for detection when the subject is upright 2. DNN FoG detection given that the subject is upright	Sensitivity (82.9%) and Specificity (97.3%)
**V.Ruonala (2013) [31]**	35 PWP and 17 patients with ET	Sample histograms during isometric contraction of biceps brachii muscle with varying loads and PCA for feature dimension reduction	Discriminate 13/17 (76%) patients with ET and 26/35 (74%) PWP

An innovative methodology for analyzing the surface EMG in PD is presented by Saara Rissanen *et al*. [30]. The system was designed referring to the analysis of crossing rate (CR) and histogram of the surface EMG signals, which are applied as the high dimensional feature vectors. CR expansion values and histogram were selected for this study due to the EMG signals that usually involve patterns of EMG bursts. The main intention of this research was due to trouble in conducting analysis for spiky impulse-like EMG waveform that usually use traditional amplitude and spectral Fourier based techniques. In this research, three types of features were applied (histogram values, values of concatenated CR expansion and histogram) for every segment of the EMG signals. Next, the dimensionalities of these feature vectors are reduced via Karhunen-Loeve transform (KLT). Lastly, study of discrimination of feature vectors will be implemented in a low dimensional eigenspace. Analysis conducted using augmented KLT applying concatenated CR expansion and histogram features had shown promising results. The proportion of accurate discrimination for the control group was 86%, while the ratio for correct discrimination achieved 72% for PWP. 

**Figure 4:**
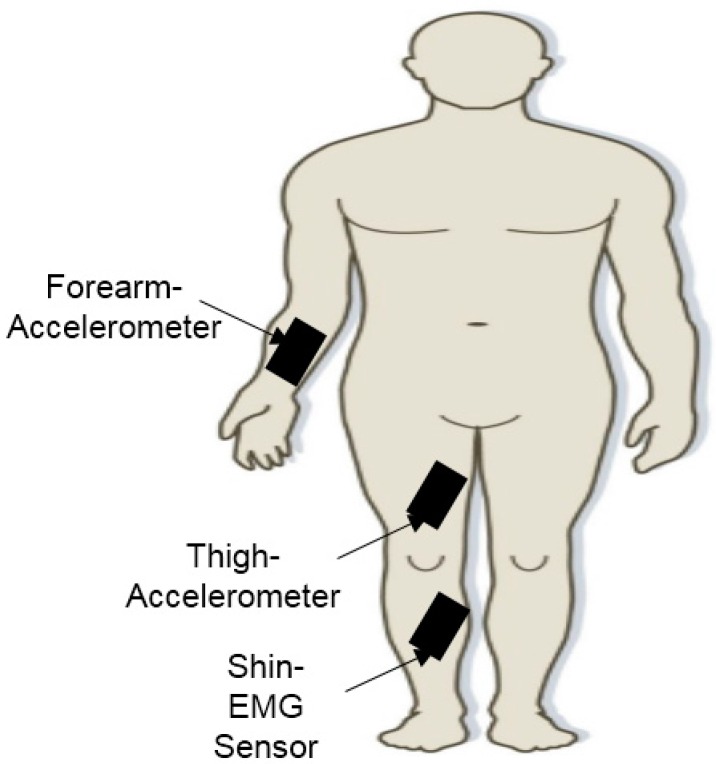
Placement of accelerometer sensors and surface EMG sensor on the subject (adapted from [34]).

Bryan *et al*. [34] described a two-phase Freezing of Gait (FoG) detection algorithm while PWP perform some daily activities that are not scheduled and restricted. This research used both wireless, wearable, miniaturized triaxial accelerometer and EMG sensor results as input features of a dynamic neural network to detect FoG instances. As shown in Figure 4, two triaxial accelerometer sensors are positioned on one side of the forearm and thigh of the patient, while one surface EMG sensor is positioned on the shin. For this study, the researchers planned and offered a two-phase algorithm: (1) Linear classifier for detection when the subject is upright either in their standing position or in walking position; (2) Dynamic neural network (DNN), which was applied for FoG detection given that the subject is upright. FoG detection can only be conducted when the subject is either trying to begin walking while in their standing position or trying to carry out walking. Thus, linear classifier was applied in the first phase of algorithm development for the detection of an upright state. After the confirmation that the subject is to be upright for more than 4 sequential seconds using the first stage classifier, the second stage was conducted by applying DNN over the break whereby the subject is in upright posture. This conveyed results with 83% sensitivity and 97% specificity upon assessing the efficiency of this system on experimentally collected datasets. 

According to research conducted by V.Ruonalla *et al*. [31], there is a difficulty in differentiating between PD and essential tremor (ET) as both of them had the possibility to occur under the same environments. The main symptoms of PD-resting tremor (4–6 Hz) and ET-postural tremor (5–7 Hz) are different but usually occur overlapping between the two. There are proofs that PWP usually have postural tremor as well as resting tremor and roughly 20% of ET patients having resting tremor. This overlapping of symptoms between ET and PD causes difficulty in classifying between PD and ET. This investigation aims to come out with solutions in order to differentiate PWP and patients with ET through EMG measurements. Data collection was conducted from the biceps brachii muscle of 17 patients with essential tremor and 35 PWP of other motor disorder during isometric tension. The preprocessing step was conducted using the smoothness priors method whereby the raw signals were high pass filtered. After filtering, segmentation of signals were done with a length of 2048 ms and 75% overlapping. Morphology analysis was then implemented using a sample histogram, which was calculated with 200 bins for each epoch due to the spiky nature of the EMG signals. Next, feature reduction using principal component analysis (PCA) was applied to ensure that the histogram could be visualized with only useful features. These three new features (*i.e*., height of histogram, sharpness of the peak and side difference) act as the new input parameters for the final classification stage. The experimental results had presented the comparison for every two features vector combination. However, among them, histogram’s height and side difference between left and right hand were the best features that show the difference of ET from PD. This technique had shown results of 76% accuracy for patients with ET and 74% accuracy for PWP [31].

### 2.2. Monitoring PD Using Electroencephalogram (EEG)

PD is a neurodegenerative disorder resulting from the death of dopamine-generating cells in the substanstia nigra, a region of the midbrain. Besides the cardinal symptoms of PD such as tremor, muscular rigidity, bradykinesia and postural instability, this motor impairment also goes along with the wide range of non-motor symptoms such as depression, decision-making dysfunctions, sleep disturbances, and autonomic deficiencies. As biological signals reflect the inherent activity of the autonomous nervous system (ANS) or the central nervous system (CNS), bio-signal captured such as electroencephalography (EEG) has been proved to provide informative characteristics in addition to be less invasive and the one with the best time resolution compared to other modalities. EEG is a recording of the electrical activity along the scalp, produced by the firing of neurons within the brain. In clinical contexts, EEG refers to the recording of the brain’s spontaneous activity, as recorded from multiple electrodes placed on the scalp. In general, EEG signals have been used in order to identify and analyze brain activity and dysfunctions relating to different neurological disorders such as PD. Evidences of such activity are reported in the majority of EEG frequency bands such as theta (4–8 Hz), alpha (8–13 Hz), beta (13–30 Hz) and gamma (30–49 Hz). To the best of the author’s knowledge, previous researches conducted using EEG data are based on emotion deficits and there is less implementation of EEG related to classic motor symptoms and signs of PD.

A technique for FoG detection using EEG signals was conducted in A.M. Ardi *et al*. [36] research using wavelet decomposition and pattern recognition techniques. FoG is characterized as a common gait impairment and ordinary cause of falling among PWP, which is one of the most disabling walking posture instabilities of PD. From the experience of the patients having FoG, they declared to have a feeling of their feet stuck to the ground and being temporally unable to initiate gait [37,38]. 4-channel wireless EEG system was applied in this study whereby the sensors were placed at occipital one (O1-primary visual receiving area), parietal four (P4-navigational movement area), central zero (Cz-primary motor area) and frontal zero (Fz-supplementary motor area). In this study, 26 PWP with significant FoG were recruited. Wavelet Energy and Total Wavelet Entropy were extracted from EEG subbands: delta (A6: 0–3.9 Hz), theta (D6: 3.9–7.8 Hz), alpha (D5: 7.8–15.6 Hz), beta (D4: 15.6–31.3 Hz) and gamma (D3: 31.3–62.5 Hz) using the multiresolution decomposition of EEG signals based on Discrete Wavelet Transform (DWT). Daubechies (db4) wavelets are chosen as the wavelet function due to their smoothing features, which are suitable for detecting changes of the EEG signals. The benefit applying this wavelet transform was to ensure excellent feature extraction obtained through the non-stationary EEG signals [39]. Next, non-parametric statistical analysis through Wilcoxon Sum Rank Test was used for evaluating the statistical differences between the extracted features. Finally, classification stage was performed using the three layers Back-Propagation Neural Network (BP-NN) classifier that has the capability to identify the onset of FoG in walking posture of PWP through the extracted features. By applying this classifier, 56% of the overall data were used as training while the remaining 25% were used for validation and the remaining 19% for testing applying the Levenberg Marquardt algorithm. Tangent sigmoid is chosen as the activation function of this classifier. Through this classifier, the results have verified the possibility of applying the EEG in the forthcoming cure of FoG detection in PD patients during walking with the highest accuracy of 76.7% using the selected features [36]. It is a challenging task in performing the feature extraction and classification of EEG signals for healthy controls and PWP whereby several signal processing methods have already been proposed for classification of the EEG non-linear and non-stationary signals. 

**Figure 5:**
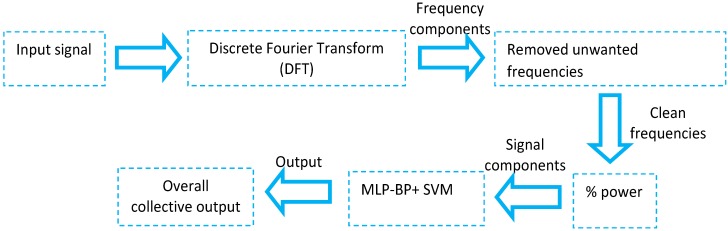
General system procedure for PD detection using EEG signal (adapted from [40]).

In the latest research, Priyanka G. Bhosale *et al*. [40] had engaged in the grouping of two classifiers: (1) Support Vector Machine (SVM) and (2) Multilayer Perceptron Backpropagation (MLP-BP) for PD detection from the background EEG signals. Figure 5 shows the overall methodology presented in this research. Useful features were extracted by applying the Discrete Fourier Transform (DFT) and the statistical features are then calculated from these clean frequency components using % power formula in order to obtain different frequency bands, which are the inputs for the classification stage. For the classification stage, SVM selects a discriminate hyper plane that maximizes the margins, which is the distances from the nearest training points for class identification. By using SVM, it enables classification with two types of boundaries: linear decision boundaries (linear SVM) or nonlinear decision boundaries using “kernel trick” (e.g., Gaussian SVM, Radial Basis Function (RBF) SVM, *etc*.). On the other side, MLP is a feed forward artificial neural network model utilizing a supervised learning technique known as backpropagation for training the network. Both classifiers were combined together instead of using a single classifier as SVM provides best training accuracy while MLP provides the best testing accuracy than others. The experimental results had shown that the combination of both classifiers had the capability to make an identification between the two classes of datasets: Healthy or Parkinson’s [40]. 

### 2.3. Monitoring PD Using Brain Imaging Modalities or 3D Motion Analysis

Dan Long *et al*. [41] presented an objective solution for PD diagnosis using non-invasive neuroimaging modality technology through the integration of multi-modal MRI. Two different types of images were collected from each subject during the data collection: (1) Non-invasive technology of resting-state functional MRI (rsfMRI) and (2) Voxel-based morphometry (structural image). The benefits of rsfMRI are having high temporal and spatial resolution that had the capability in studying the abnormal function of the brain in PD. While for structural images, voxel-based morphometry had the ability to conduct analysis on the changes of the structural brain from the entire brain viewpoint. Integration of both imaging modalities had the ability in distinguishing early PWP from healthy population and correlate with the severity of PD. Template based approach was used as the feature extraction techniques whereby for rsfMRI data, multi-level characteristics at different levels were extracted: (1) Regional Homogeneity (ReHo); (2) Amplitude of Low Frequency Fluctuation (ALFF); and (3) Regional Functional Connectivity Strength (RFCS). While, for structural images, Gray Matter (GM), White Matter (WM) and the Cerebro-Spinal Fluid (CSF) volume characteristics were extracted. In this research, feature selection was conducted using two-sample t-test due to decrease of recognition rates of certain extracted features and the generalization of noise. By applying this test, only the features with significant difference (*p-value* < 0.05, uncorrected) were selected when comparing the feature values of various brain regions for both PWP and healthy controls. Finally, SVM was chosen as the supervised machine-learning classification algorithm using the leave-one-out cross validation technique that produces overall best accuracy of 86.87%, sensitivity of 78.95% and specificity of 92.59% [41]. 

As the resting tremor is one of the primary motor symptoms of PD, research had also been conducted by Magdalena *et al.* [42] using multimodal motion capture (MOCAP) system for registration of 3D positions of body markers, ground reaction forces and EMG signals to collect kinematic measurements of upper limbs. In this work, the data collection was conducted under four circumstances where the stimulator was turned ON/OFF and medication was ON/OFF. For the preliminary stage, the analysis begins with assessing the signal background to remove the trend component and constant from the triaxial coordinates of each signal using the recursive histogram algorithm from both right and left markers. Next, Euclidean distance between the actual position of the marker and its centroid (rooted sum of squares: RSS) was calculated to obtain two 3D tremor signals that correspond to the left and right markers. In addition, frequency analysis was carried out using Fast Fourier Transform (FFT) where the calculation of amplitude spectra was done. Based on this, the maximal amplitude, mean amplitude and area under the curve of the spectrum in the range of 3–7 Hz and 4–6 Hz were calculated. Lastly, statistical analysis was conducted applying t-test where the obtained results had shown the occurrence of statistically significant differences in certain tremor parameters between different tremor conditions [42,43].

### 2.4. Monitoring PD Using Wearable Sensors

#### 2.4.1. PD Symptoms Assessment-Tremor and Bradykinesia

**Figure 6:**
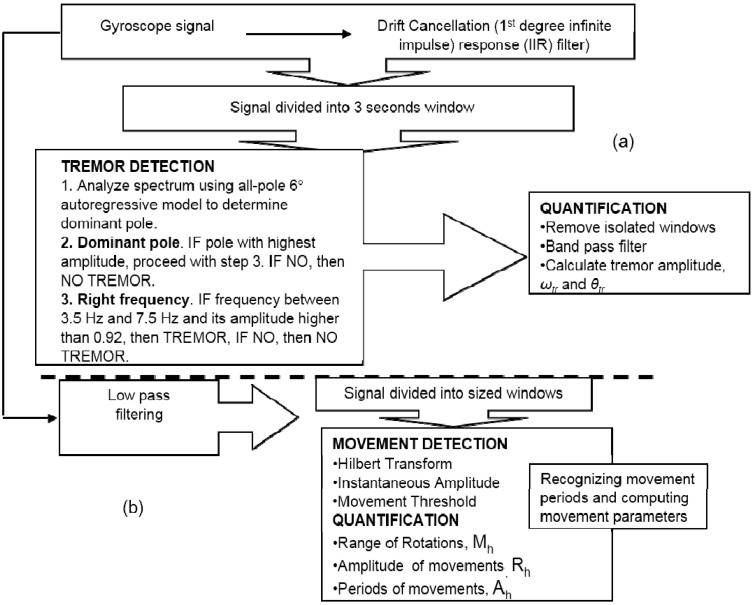
Flowcharts of the techniques. (**a**) Detection and quantification techniques for tremor; (**b**) Techniques for bradykinesia quantification.

In research conducted by Arash *et al*. [44], quantification of tremor and bradykinesia among PWP was presented. For the first study, research was conducted on 10 PWP and 10 healthy controls. Each of them was required to perform a list of activities, whereby each of the task signifying usual daily life activity. The measurement system includes three miniature uniaxial gyroscopes, which functions to measure the angular velocity during the movement of the forearm in three directions: roll, yawn and pitch. During the second study, data collection was conducted using a newly designed system that is integrated with two gyroscopes. However, only 11 PWP upper extremity movements were recorded continuously for several hours whereby they were allowed to carry out any of their daily activities. The range of the gyroscope (Murata, ENC-03J) used for this research after calibration was ±1200°/s with a weight of 35 g. To record the signals during each measurement, a light, portable data logger (Physilog®, BioAGM, CH, Switzerland) with 8 MB memory card and a sampling rate of 200 Hz and 12-bit resolution of A/D was carried by the subject. The proposed new algorithm to detect tremor and bradykinesia had been validated through the obtained sensitivity and specificity results. Figure 6 shows the flowchart of the overall methodology for quantifying tremor and bradykinesia [44]. For the first study, average sensitivity of 99.5% and specificity of 94.2% was obtained through the proposed algorithm for tremor detection in contrast with the video recording. In addition, it was also found that the parameters related to the estimated tremor (tremor amplitude, ω*_tr_* and θ*_tr_*) and bradykinesia (M_h_, R_h_ and A_h_) show high correlation towards UPDRS tremor and bradykinesia subscore. Correlation between the UPDRS tremor subscore and tremor amplitude and correlation between UPDRS bradykinesia subscore and bradykinesia parameters (M_h_, R_h_ and A_h_) were studied. A high correlation between the parameters related to tremor and bradykinesia and UPDRS subscore was achieved using Pearson’s correlation and Partial correlation (for eliminating the effect of ON/OFF factor) whereby feature with *p*-value above 0.05 were considered as non-significant [44,45]. 

#### 2.4.2. PWP Physical Activities Monitoring

In the same year, Arash *et al*. [46] also propose a new ambulatory method to monitor the physical activities of PWP using a convenient data logger with three bodies-fixed inertial sensors consisting of one gyroscope and two accelerometers, which had capability of continuous recording. The subjects involved in this research consist of ten PWP that had undergone deep-brain stimulation of the subthalamic nucleus (STN-DBS) treatment and ten healthy controls. The patients were requested to perform a list of typical daily life activities and the whole process was recorded. Two gyroscopes (Murata, ENC-03J, ±600°/s) were placed on the shanks for walking posture detection while a gyroscope (Murata, ENC-03J, ±200°/s) and an accelerometer (Analog Device, ADX202, ±2 g) was placed on the trunk for lying posture detection. The signals from the sensors were recorded on a light, portable data logger (Physilog, BioAGM, CH, Switzerland) with a sampling frequency of 200 Hz. The main aim of conducting this research was to classify PWP basic posture allocation such as walking, lying, sitting and standing durations. Additionally, this research also aimed for the detection of Stand-Sit (StSi) and Sit-Stand (SiSt) transitions whereby several parameters of transitions pattern were extracted as described in Table 2. The detection and separation of SiSt and StSi transitions from other body movements were obtained based on the calculation of trunk movement kinematic features during transitions between standing and sitting postures and by using two statistical classifiers based on the regression model. This model is an effective method for making discrete outcome predictions, for instance, transition *vs.* non-transition deprived of any particular distribution assumptions. The first classifier goal were to allocate each patient a possibility of being a transition *vs.* being a non-transition, while the second classifier aimed at separating the SiSt and StSi transition. Finally, information about transitions and activities performed before and after were used by a fuzzy classifier for detecting the periods of sitting and standing. The accuracy of the proposed technique has been determined by comparing with the video recording that is examined by UPDRS score experts. Through this research, the detection of the basic body position allocation had shown high specificity and sensitivity, both in healthy controls and PWP. Significant dissimilarities are also clearly seen in parameters related to SiSt and StSi transitions between PWP and healthy controls and between PWP with and without STN-DBS turned on. The classification of the basic activities, *i.e*., walking, standing, siting and lying had a sensitivity of 85%, 83.6%, 86.3%, and 91.8%, respectively, and specificity of 97.8%, 96.5%, 98.0%, and 99.8%, respectively. While for detection of StSi and SiSt transitions, the proposed algorithms had shown 83.8% sensitivity and a positive predicted value (PPV) of 87.0% [46,47].

**Table 2:** List of posture transition related parameters [46,47].

Parameter	Description
***TD* (*s*)**	Period of transition: Time break between the two positive peaks before and after the transition time in the trunk tilt, *θ_g-lp_* signal
***Min(θ_g-lp_*) (*°*)**	Minimum amplitude of negative peak of flexion and extension tilt of the trunk that in general much higher in the real posture transition patterns compared to the non-transitions patterns
***Max*(*α_trunk-lp_*) (*g* × 10^−3^)**	Signal α_trunk-lp_ was produced through the norm of the acceleration vector measured by the perpendicular accelerometers of the trunk sensor filtered using a low pass filter. The maximum, minimum and range, of this signal were generally higher in the posture transitions and lower in non-transitions. The relative time of the minimum and maximum peaks of this signal compared to the transition time was also different between SiSt and StSi transitions.
***Min*(*α_trunk-lp_*) (*g* × 10^−3^)**	
***Range*(*α_trunk-lp_*) (*g* × 10^−3^)**	
***T*[*Max*(*α_trunk-lp_*)] (*s*)**	
***T*[*Min*(*α_trunk-lp_*)] (*s*)**	
***Range*(*θ_g-lp_*) (*°*)**	Range of flexion and extension tilt of the trunk where the value of this parameter was lower for the non-transitions than for the real posture transitions.

#### 2.4.3. Levodopa Induced Dyskinesia (LID) Detection in PD

Research conducted by Keijsers *et al*. [48] had demonstrated the highly effective use of neural networks for detection of dyskinesia and differentiating dyskinesia from voluntary movements. Measurement of PWP were continuously conducted in a home environment via six triaxial accelerometers (ADXL-202; Analog Devices, Norwood, MA), which were positioned at different parts of the body while PWP completed roughly 35 everyday life activities. The accelerometer signals were recorded using a recorder (Vitaport 3, TEMEC instruments, Kerkrade, The Netherlands) with a sampling frequency of 64 Hz. Neural network using MLP was trained to measure the severity of LID with numerous accelerometer signal features as shown in Table 3. A comparison was made between the scores obtained through MLP, specified by a linear transfer function reflecting the Abnormal Involuntary Movement, scale (AIMS) score and the validation by physicians, who made the evaluation of the PD patient’s condition through video recordings. Thirteen variables were derived from the raw accelerometer signals for every 1-min interval before being passed to the classification stage. These variables function as the input for the classifier while the output will be the rating scores obtained through the physicians. Classification through neural network was reflected as correctly classified if the output of the neural network and scores obtained through the clinicians was ≤0.5. The proposed neural network classifier successfully differentiates dyskinesia from non-dyskinesia with the recognition rate of 93.7% for arm, 99.7% for trunk, and 97.0% for leg [48,49].

**Figure 7:**
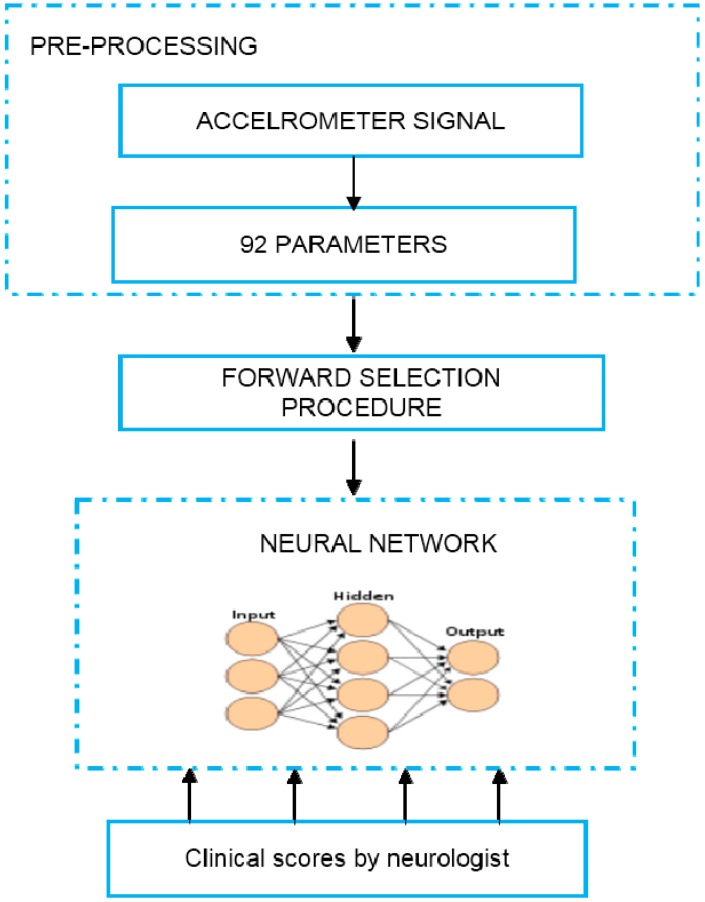
An overview of the methodology of dyskinesia severity assessment.

In addition, Keijsers *et al*. [50] also conducted another research that focused on the design of the trained neural network and the role of important features extracted from the raw accelerometer signals, which are used as input variables for an accurate detection and ratings of dyskinesia. The objective of this research was to investigate the performance of an ideal neural network for dyskinesia detection and rating. Table 3 showed the list of parameters used and their description. Figure 7 shows the schematic block diagram of the data preprocessing and successive neural network approach in dyskinesia severity assessment. For every part of the body, the dyskinesia severity might be not similar that made the reason of evaluating it for every body part individually. This is also because the variables required are different for detecting dyskinesia in each body part. The forward selection procedure was used to determine the order of the important parameters and searching for the best features to be used as the input for LID classification. The benefit of this step is that it only includes variables, which bring improved performance without the need of any earlier information and restriction. From the analysis done, the most significant parameter for dyskinesia detection was the percentage of time during trunk/leg movements and standard deviation of less dyskinetic leg segment velocity. The remaining movement features are also significant, but that applies in a different way to other parts of limb segments. For instance, the grouping of percentage of time during wrist movements, and the percentage of time during the subject is in the sitting posture, described the major part of the variance of the output for arm [21,48,49,50,51].

**Table 3:** List of input parameters for neural network [50].

Variables	Description
**$\bar{V}$ segment**	Mean of segment velocity
**$\bar{V}$_<3 Hz_ segment**	Mean of segment velocity for frequencies below 3 Hz
**$\bar{V}$_>3 Hz_ segment**	Mean of segment velocity for frequencies above 3 Hz
**$\bar{V}$*_<_*_3 Hz_ segment/$\bar{V}$_>3 Hz_ segment**	Ratio between $\bar{V}$_<3 Hz_ segment and $\bar{V}$_>3 Hz_ segment
**SD (*V*) segment**	Segment velocity standard deviation
**% *V_θ_* segment**	Percentage of time of segment’s movement
**$\bar{V}$_θ_ segment**	Mean segment velocity of segment’s movement
***P*_1–3 Hz_ segment**	Power for frequencies in the range between 1 and 3 Hz
***P*_<3 Hz_ segment**	Power for frequencies in the range below 3 Hz
**$\bar{\text{ρ}}$_segment-segment_**	Mean value of the normalized cross-correlation between the segment velocities of different segments
**Max (ρ_segment-segment_)**	Maximum value of the normalized cross-correlation between the segment velocities of different segments
**% sitting**	Percentage of time during subject sitting posture
**% upright**	Percentage of time during subject upright posture

#### 2.4.4. Estimation of PD Symptoms Severity-Tremor, Bradykinesia and Dyskinesia

Patel *et al*. [52] presented results from a preliminary study for estimation of symptoms, severity and motor complications in PWP through the assessment of accelerometer data. In this research, SVM classifier was chosen as the classifier for tremor, bradykinesia and dyskinesia severity estimation using the data features obtained through the accelerometer. Twelve subjects were diagnosed with idiopathic PD of stages 2 and 3 were recruited whereby their age ranged from 46 to 75 years. The PWP were asked to accomplish a list of fixed motor tasks developed clinically for PD evaluation where these tasks are part of the activities in UPDRS that includes finger-to-nose (reaching and touching a target), finger tapping, repeated hand movements (opening and closing both hands), heel tapping, quiet setting, and alternating hand movements (repeated pronation/supination movements of the forearms). Comparisons were made between the data collected with the clinical scores obtained through visual inspection of video recordings. The intention of conducting this research was taking concern regarding the design of signal procedure techniques that can be applied to the nodes of the body sensor network, hence decreasing data volume to be transmitted from the network nodes to the base station. Analysis was conducted on the outcomes were achieved through the accelerometer data in order for assessing the consequences on the estimation of clinical scores. This includes the length of the window utilized that ultimately compute into data features, use of various SVM kernels (polynomial, exponential and radial basis kernel) and use of data features (data range, cross-correlation-based value, rms value, frequency based features and signal entropy) derived from different motor tasks. In addition, analysis was also done for assessing the types of combinations of data features that carried useful information, which may be reliable to assess the PD symptoms severity and motor complication. Table 4 provides the list of criteria studied for optimizing the algorithms by reducing the error, which will affect the estimation of clinical scores for measuring the symptom severity [9,22,25,52]. The analysis presented that window length of 5 s is optimal while the third-order polynomial kernel was selected to be desirable due to smaller misclassification value. Results also indicate the possibility of using the three feature types (rms value, data range value and two frequency-based features, *i.e*. dominant frequency and the ratio of the energy of the dominant frequency component over the total energy) achieving average estimation error values of 3.4% for tremor, 2.2% for bradykinesia, and 3.2% for dyskinesia [52].

**Table 4:** List of criteria considered in estimating symptom severity of PD [52].

Criteria	Description
**Length of the windows**	Used for selecting data segments of the accelerometer data and deriving data featuresAchieving the average estimation errors below 5% Utilized length of windows ranging from 1 to 7 s with an increment of 1 s
**SVM kernels**	Three different types of kernels: polynomial, exponential and radial basis
**Feature types**	Five features types were compared: Data range, root mean square (rms) value, cross-correlation-based features, frequency based features and signal entropy

#### 2.4.5. PWP Home Monitoring System Using Web Based Application

**Figure 8:**
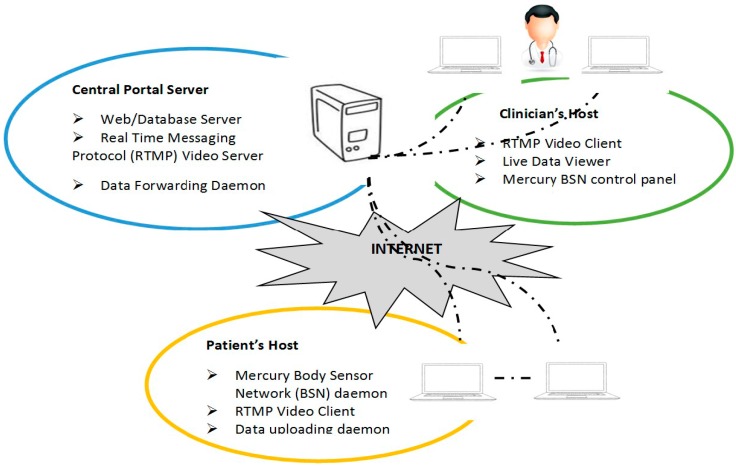
A general idea of the design of the home monitoring system (adapted from [53]).

In the following year, Patel *et al*. [53] conducted his research toward the home-monitoring development for PWP who experience severe motor fluctuation using a wireless wearable sensor, whereby information is transmitted to the clinicians through an application using web-based. The implemented home-monitoring system comprises software services, conducting at three tiers: central portal server, patient’s hosts and clinician’s host as shown in Figure 8. The well-provisioned central portal server was in charge of providing a protected and trustworthy central location via coding services in order to coordinate the actual data collection and video services, ensuring data safety and high accessibility of the remote health monitoring service. The patient’s host (laptop) mostly occupies at the particular PD patient’s home and runs the body sensor network (BSN) platform that functions to collect motion data and continual upload motion data to the folder created in the central portal server. Lastly, the clinician’s host occupies in clinic only required Internet access to use the service. Besides that, this system also provides the capability of video interaction between the patients and the clinicians. This research implemented a web-application known as MercuryLive, which comprises a graphical user interface for displaying the motion signals together with the video conference, allowing clinicians for viewing and data annotation throughout every data collection session. Data collection using this developed system will then be processed in order to come out with an estimate of clinical scores that measure the symptom severity and motor complications of PWP using the algorithms for wearable sensor data analysis. In this work, a combination of web-based application together with the technology using the wireless wearable sensor for home monitoring had provided trustworthy quantitative information, which is useful in gathering clinically relevant information for PWP management and also for clinical decision making [53].

Chen *et al.* [54] conducted further studies on the home monitoring system, MercuryLive that offers an integrated platform, ensuring assessment of data collection from PWP gathered with wearable sensors through the web application. As mentioned previously in [53], this system offers the clinician opportunity in interacting with PWP in their homes, configuring the sensor nodes for hand application and recording annotated data. The advantages of data collection in the home environment using this system are having the capability for allowing the clinicians to expand the excellent care of PWP and at the same time reducing costs. The focus of this study is on the characterization of the system as the system design and implementation had previously been mentioned by Patel *et al.* [53]. The characterization of MercuryLive was made by assessment of latencies and bandwidth requirements at different tiers (central server, patient host and clinician host) of the system. The latency time is the time between the packet delivery time from the sensor and the packet receiving time by the clinician. Latency calculations were created according to the timestamps taken at each host as relevant data packets arrived at each tier of MercuryLive that was further described in Table 5 [54]. The system clocks for every single host were coordinated into similar server- Network Time Protocol (NTP) to ensure the measurement accuracy. The preliminary testing results show the suitability for monitoring PD’s patients in the home environment and the gathering of information to assist the titration of medication. It revealed an average data latency of less than 400 ms and video latency of about 200 ms with the video frame rate of approximately 13 frames per seconds when 800 kb/s of bandwidth were accessible using 40% video compression [53,54].

**Table 5:** Different types of latency and their description [54].

Latency	Description
**Command Latency**	▪ Time between commands delivered to the clinician’s host and the patient’s host received and acknowledged. ▪ Commands are delivered by the clinician’s to execute configuration on the body sensor network such as changes in data sampling frequency
**Data and Video Latency**	▪ Data latency: Interval in live streaming of that decimated version of the sensor data ▪ Video latency: Time between generation of a frame at the patient’s end and the appearance of the frame at the clinician’s end
**Recovery latency**	▪ Time needed for the system to begin re-operate after the miscarriage of the system ▪ Latency values were predictable as the alteration between the two timestamps linked with the restart command and with the finishing point of the re-initialization process of the sensor node
**Data upload latency**	▪ Logging of the raw data or the data features into the onboard flash memory and uploading into the central server when possible

#### 2.4.6. Assessment of Gait Impairment in PD

Research conducted by Cancela *et al*. [55] concentrated on the design of non-supervised methods for the gait impairment assessment in PWP. Detection of motion changes was done using five triaxial accelerometers-ALA-6g (ANCO, Athens, Greece) placed on the limb and one triaxial accelerometer-ALA-6g (ANCO, Athens, Greece) on the belt of PWP. The development of this system for PD symptoms long duration monitoring permits physicians to distinguish the intakes of the medication and subsequently shows improvement in the patient’s reaction towards the cure. Besides that, this allows long term continuous monitoring of gait rhythm and other gait parameters in order to achieve assessable estimations of motor fluctuations in everyday life activities and assessment of the influence of medication on different gait parameters. The first step of this research was observing the recorded signals PWP and healthy control moving freely and performing their daily activities. By contrast, motor signals from PWP display an important distance compared to healthy control patterns. There are various ranges of features that can be connected to healthy pattern gait through spectrum analysis, which aids to create a contrast with the output of PWP recording. Some measures (*i.e*., step frequency, stride length, entropy and arm swing) related to magnitudes have been defined, based on the overview of both signals. Results shown that the use of measures like entropy and arm string were the better choice showing significantly better performance to provide a comprehensive and precise status of the gait impairment [55].

#### 2.4.7. Detection of PD Motor Symptoms: Uncontrolled Home Environment

**Figure 9:**
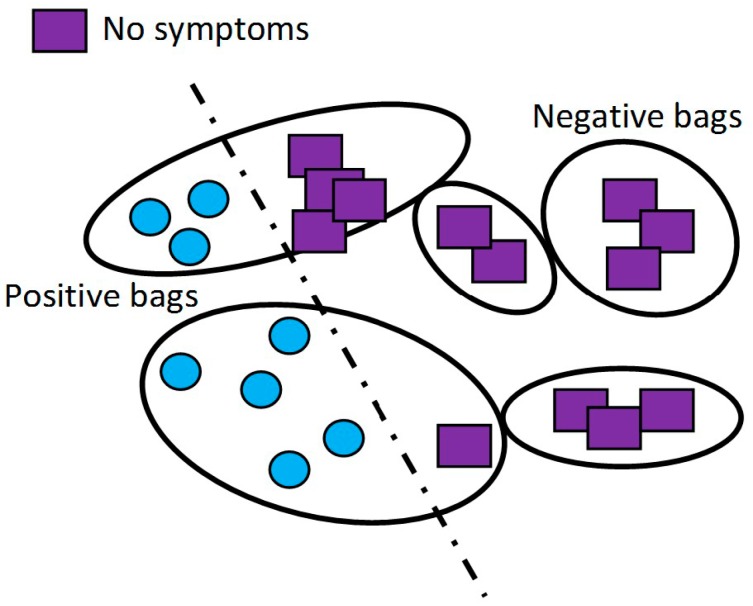
The intuitive idea behind multiple instances learning (MIL) (adapted from [59]).

Samargit Das *et al*. [56] conducted his research for PD motor symptoms automatic detection in everyday life environments by applying weakly supervised learning context called multiple instances learning (MIL). Adapting and training supervised learning classifier was challenging due to lack of reliable ground truth information when monitoring under uncontrolled environment. On the other hand, for each time instant, this MIL algorithm only requires knowing the existence of symptom pattern anywhere within the time interval without knowing the exact existences of the symptoms. The labels will be allocated into bag of cases (*i.e*., feature vectors collection over a time period) whereby the bag will be denoted as positive bag if a minimum of one case inside the bag is true, and the bag is denoted as positive bag if the entire cases inside it are all false (referring Figure 9). MIL will automatically learns those patterns by detecting the time period of symptoms existence as well as identifying cases of the symptoms [56,57,58,59,60]. 

This research had shown promising preliminary results using five 3D accelerometers placed at the waist and limbs. Several features that include high frequency energy content, correlation and the frequency domain histogram were implemented in this research. MIL algorithms play a part in looking for an axis-parallel hyper-rectangle (APR) in the feature space, which captures the target concept. The authors proposed solutions to this algorithm recommended three variants of the APR algorithm: (1) a “standard” algorithm; (2) an “outside-in” algorithm; and (3) an “inside-out” algorithm. However, in this research, they implemented an iterative, discriminative variant of the APR algorithm (ID-APR) for MIL. This is an integration between the standard and outside-in APR algorithms. Comparison between the performance of ID-APR and other MIL algorithms such as multiple instance SVM (MI-SVM), citation k-nearest neighbor (KNN), diverse density (DD) and expectation maximation version of DD (EM-DD) were conducted. The performance characteristics of the algorithm were analyzed through the computation of their total bags of correctly classified as symptoms percentage whereby ID-APR based MIL algorithm performs better compared to the rest of the algorithm with over 90% accuracy [56].

#### 2.4.8. PD Hand Tremor Monitoring

LeMoyne *et al*. [61] demonstrated the use of wireless accelerometer for monitoring PD hand tremor. The experimental setup of this research consists of two wireless accelerometer nodes that were tandem activated. The first wireless accelerometer represents the control, which was placed in a static position and second tandem activated wireless accelerometer was secured by a glove to the dorsum of the hand that simulated PD hand tremor. The triaxial accelerometer data collected were wirelessly sent to a local PC for post-processing. Based on the three orthogonal acceleration components, the magnitude of the acceleration vector was calculated to represent the acceleration waveform for the temporal domain. The acceleration waveform of 5-s duration between 2.5-s and 7.5-s of the 10-s acceleration waveform sample was analyzed to reduce transient effects. The time-averaged acceleration technique using a trapezoid rule was acquired for quantifying each respective acceleration waveform sample of both the simulated PD tremor and static condition, bound by a 98% confidence level based on a 2% margin of error about the mean. Statistical study using one-way ANOVA with alpha <0.05 conducted exhibits statistical significance during the comparison of time averaged acceleration between the static positioned wireless accelerometer node and simulated PD hand tremor wireless accelerometer node. This configuration incorporating the wireless accelerometer application has demonstrated a significant degree of accuracy, consistency, and reliability for quantifying acceleration waveform of simulated PD tremor [61,62,63,64].

#### 2.4.9. Detection of Freezing of Gait (FoG) in PD

FoG commonly occurs on PWP during the advanced stage of PD associated with the disease duration and severity, representing common reason of falling and consequent injuries in PD patients, which significantly impairs quality of life. FoG had relations with falls is the transient block of movement that is triggered by gait, which appears most often during turns, before initiating gait, in tight quarters such as doorways, while negotiating an obstacle and in stressful situations. Based on the analysis conducted by Macht *et al*. [65], 47% of the total 6620 PD patients reported regular freezing whereby 28% of them experienced FoG daily. This symptom had obstacles in predicting the occurrence and sensitivity to external factors such as environmental triggers, cause difficulty in detecting FoG under laboratory or clinical condition. The cause of freezing is unclear, but the condition of the patients becomes serious once they get nervous, in places surrounded by many people, while crossing doorways, in elevators and restricted areas or sudden change in their walking patterns. Although previous researchers have proposed that longer time span of dopaminergic treatment is correlated with FoG, the progression of the PD alone may be responsible for the development of FoG. It is important to have good results in both the severity and effect of FoG detection and rating. In order to come out with successful management of PD patients with FoG, it is required to have attentive assessment and gait pattern analysis [66,67,68,69,70,71,72,73,74,75,76].

In Haritz *et al*. [75] research, he had proposed a method for freezing detection in PWP using gait analysis in the frequency domain. This pilot study used an accelerometer to measure the acceleration in 3 axes and a gyroscope to compute angular velocity in 2 axes in three locations of the lower extremities for monitoring foot, shank and thigh movements. Sparkfun Inertial Miniature Unit (IMU) sensors were used to obtain kinematic data of each point, which incorporates the new IDG300 dual-axis gyroscope and the Analog Devices triple axis ADXL330 accelerometer providing 5 axis of sensing (Roll, Pitch, X, Y, Z). IDG300 is an integrated dual-axis gyroscope with integrated X- and Y-axis gyro on a single chip; full scale range of 500/s; integrated low-pass filters and the ADXL330 is a 3-axis accelerometer of a minimum full-scale range of 3 g. The intention of this research was applying the frequency domain analysis in determining the most suitable sensor position and selection of signal’s sensor that is most appropriate for FoG detection. Several parameters had been taken into consideration in order to conduct this analysis; the dominant frequency, power spectral density (PSD) Quartiles, power above and below the dominant frequency and the freeze index (FI). After data collection, the resulting signals were processed using various spectral analysis techniques such as FFT that transform the broken overlapping frame into the frequency domain for obtaining the PSD for each frame. Changes of the PSD towards higher frequency will be interpreted as FoG as the power of the signal above this frequency increase during FoG episodes. Results shown that sensor placement at the lower limbs (80.8% of FoG episodes detected correctly) and heel perpendicular to the coronal plane (82.7% of FoG episodes detected correctly) with a sensing magnitude of the angular velocity and acceleration respectively shown the best classification variables [75].

Research was also conducted by Moore *et al*. [74] that acquires vertical linear acceleration of the left shank. Detection of FoG in PWP was conducted by means of an ankle–mounted sensor array during a predefined walking and standing test where the data were transferred wirelessly to a pocket PC at a rate of 100 Hz. The sensor consists of an IMU, 9 V battery and Bluetooth serial transmitter that weight less than 130 g. The frequency spectrum obtained from the accelerometer were analyzed, where the power analysis showed that FoG go together with high frequency components of leg movement in the 3–8 Hz (freeze band) compared to the 0.5–3 Hz (locomotors band). Results obtained allowed the calculation of the FI, which was calculated as the power in the freeze band divided by the power in the locomotors band. The value of FI will be relatively stable during PWP normal movement, but increases due to gait freeze whereby a threshold value is chosen such that the value of the FI above this limit would be considered as FoG events [67,73]. This global threshold had shown to be 78% accurate in detecting FoG events before being customized to PD patients [74].

### 2.5. Monitoring PD Using Audio Sensors

Recent studies had also shown interest in the link between PD and speech deficiency. Lately, varieties of speech signal algorithms that had aimed for the PD symptom severity detection have been presented. Vocal impairment relevant to PD is among the initial indicators with studies reporting approximately 70%–90% prevalence. The symptoms existing in speech disorder include loudness reduction, vocal tremor increment, and breathiness (noise). Voice deficiency related to PD is termed as dysphonia (incapability of producing normal vocal sounds), hypophonia (reduced voice volume), and dysarthria (difficulty in pronouncing words). Speech disorders have been linked to PD, and there is solid supported proof of performance degradation in voice related to PD progression [77,78,79,80,81]. The degree of vocal deficiency is frequently assessed through sustained vowel phonation or running speech. However, the use of sustained vowels cannot capture some of the voice impairment that can be found in running speech, for instance, the integration between vowels and consonants, while the use of running speech can be categorized as a more convincing impairment test. On the other hand, analysis conducted through running speech may be more complicated because of the articulatory and other logistic confusions. By applying phonation test conducted through sustained vowels, it had the ability to provoke dysphonia symptoms. In addition, detection of dysphonia can be best efficiently accomplished without confusing effects of articulatory or linguistic modules of running speech. Hence, it has become a general protocol of using sustained vowels whereby the subject is asked to withstand the phonation for as long as possible, making a strength to sustain stable frequency and amplitude at a relaxed level [77,78,82,83,84,85,86,87,88]. 

Studies have presented that the sustained vowel “/a/” is adequate for numerous voice assessment applications, which include prediction of PD status [85,86,89] and monitoring of PD symptoms [84,90,91,92,93]. Studies of speech disorders in term of PD have provoked the progress of many speech signal processing algorithms, for example, dysphonia measures whereby these measures had been suggested as a trustworthy tool for PD detection and monitoring [86,87,88]. For the past few years, several studies have been conducted using the available M.A.Little PD dataset from the UCI machine learning repository and achieve high success rates [87,94,95,96,97,98,99,100,101,102]. For instance, Kemal Polat *et al*. [102] had presented a comparative study by applying fuzzy c-means clustering and the experimental results had shown a maximum accuracy of 96% using k-nearest neighbor (KNN) classifier. In research proposed by H.L Chen *et al*. [101], the researchers had presented an effective diagnostic system for diagnosing PD using fuzzy k-nearest neighbor (fKNN). The analysis had been further improved where PCA had been applied in order to construct the most discriminative features set. The experimental results using the proposed fKNN classifier had shown greater performance when compared with the SVM classifier with best classification accuracy of 96% with average 10 fold cross validation method. Zahari *et al*. [100] employed the feature selection method based on Analysis of Variance (ANOVA) together with the MLP neural network (MLP-NN) classifier to predict PD. This research describes the analysis on the MLP-NN according to two types of training algorithms, which are Levenberg-Marquardt (LM) and Scaled Conjugate Gradient (SCG). Using the PD dataset by Little *et al*. [85,86,89], the classification accuracy of above 90% was achieved after applying feature selection using LM algorithms while SCG algorithms obtained accuracy above 85% after the implementation of ANOVA as feature selection [100].

Max A. Little and his co-researchers had extracted different types of features for the objective analysis of voice signals for classifying PWP from healthy controls [85,86,89]. In one of their works [86], they had conducted a remarkable assessment using present traditional and non-standard measures to classify healthy controls and PWP dysphonia detection. This investigation has presented a new dysphonia measure, known as the pitch period entropy (PPE) that is useful for several confounding effects, which cannot be controlled such as noisy acoustic surroundings a difference in voice frequency. The experiment was tested on 31 subjects, of which 23 of them were PWP. In the correlation filtering stages, ten highly uncorrelated dysphonia measures were selected. A combination of four optimal dysphonia features (HNR, RPDE, DFA and PPE) through a pre-selection filter removes redundant measures, followed by an exhaustive search gives the best overall accuracy of 91.4% by applying SVM radial basis kernel function. The results found that the combination of nonstandard techniques and the traditional harmonics-to-noise ratios had the greatest ability to separate healthy controls from PD patients [86,89]. 

Tsanas *et al*. [92] conducted studies on disordered voices of PWP whereby the research shown that the prospective for recognizing subtle differences in PD symptoms can be considerably improved through the transformation of simple logarithmic of the dysphonia measures. The speech recordings of sustained vowels from 52 PWP were chosen as subjects of this study. The recording was performed by the patients at home using a telemonitoring device. In this test, PWP were requested to withstand the vowel “/a/” sounds for as long as possible. The primary objective of this study was for the computation of the progress in UPDRS estimation exclusively depending on the log-transformation of classical dysphonia measures. Bayesian Least Absolute Shrinkage and Selection Operator (LASSO) linear regression is performed for reducing the amount of measures that was selected as features and determination of whether the log-transformed classical measures overtook the non-transformed measures. The efficiency of improvement in this developing application of programs characterization of PD symptom evaluation from voice signals, rated based on UPDRS was revealed [91,92,93]. Tsanas *et al*. [85] also conducted studies in testing the accuracy of novel algorithms that can be applied for differentiating healthy controls from PWP. In this study, current available database from the National Centre for Voice and Speech (NCVS) taken from 43 subjects (10 healthy controls and 33 PWP) were used, which involves six or seven sustained vowel “/a/” phonation from each subject. Comparison between four different efficient feature selection algorithms was conducted: (1) LASSO; (2) minimum redundancy maximum relevance (mRMR); (3) RELIEF; and (4) local learning-based feature selection (LLBFS). These dysphonia measures have selected four parsimonious subsets mapping to a binary classification response applying two statistical classifiers: random forests and SVM. Results validation shows that selected new dysphonia measures can overtake the current existing outcomes, whereby the results reach nearly 99% of classification accuracy by only applying ten dysphonia features [85,86,89].

Douglas *et al*. [103] conducted a research measuring instability in the phonatory signal by identifying disordered patterns that have possibilities to exist in voice of some PWP using nonlinear dynamic analysis and perturbation analysis. 1-second segmentation of sustained phonation was done before proceeding to the analysis stage. Nonlinear dynamic analysis will provide corresponding information for perturbation analysis, allows the combination of both analyses. This will improve the ability in describing pathological voices from PD and at the same time aiding the diagnosis of laryngeal pathologies from PD. Correlation dimension, jitter, and shimmer parameters of the acoustic signal were applied for comparing continuous vowel formations of PWP with the healthy controls. The parameters obtained through both analyses were then undergoing statistical analysis for analyzing the results obtained. The overall comparison between the control subjects and PWP was obtained through Mann-Whitney rank sum tests for each parameter. Results showed that the overall PWP have significantly higher correlation dimension values compared to healthy controls (P = 0.0016), which specify greater signal difficulty in PD vocal pathology. However, alterations in the evaluation of these two groups were significant in jitter (P = 0.014) but non-significant in shimmer (P = 0.695). The overall results showed that the combination of nonlinear dynamic analyses and perturbation analysis was necessary, which can be a representation as a beneficial technique in the research of PD vocal pathology, adding to the traditional techniques of voice analysis [103].

## 3. Discussion and Conclusions

It is essential for the medications to be optimally adjusted in order for PWP to function at their best whereby the clinicians in charge are compulsory to have a precise image of the way PWP symptoms fluctuate throughout their everyday life activities. Lately, PD cannot be handled through medication, although it offered significant improvement of symptoms, particularly at the primary stages of PD. Yet, appropriate identification at an initial stage can produce significant lifesaving outcomes [104,105]. In these conditions, the conventional methods such as patient’s subjective self-reports and patient diaries are normally not very precise and have shortcomings. Although several rating scales that were plotted to UPDRS had been designed and used by physicians, they still possess some limitation whereby UPDRS assessment is subjective, time consuming task and sensitive to inter-rater variability. Many PWP will thus be extensively reliant on clinical involvement, but physical appointments to clinic for checking and treatment are demanding for many PD patients [24,26,27]. This matter is currently a tedious challenge that the physicians are fronting when handling long durations of PD as the medical care towards these patients is increasingly complex and expensive. 

Over the past decades, researchers have devised several non-invasive, objective methods for detecting early symptoms of PD using physiological biomarkers, including EMG [29,30,31,32,35] and EEG [36,40] signals, brain imaging methods (CT scans or MRI) [39,40,41], speech difficulties using audio sensors [41,42] and wearable sensors [44,45,50,51,52,53,54,55,56,62]. EEG is a tool that is used to measure the electrical activity generated in the brain, which opens a window for exploring brain functioning and neural activity. It is a completely non-invasive technique measured using several electrodes located on the subject’s scalp, which records the electrical impulses generated by nerve cells from the brain (brain waves). In medical environments, EEG refers to the recording of brain’s unstructured electrical activity over a long time period, usually 20–30 min that includes preparation time, as recorded from multiple electrodes located on the scalp [106]. Even though current EEG technology can precisely identify brain activity at resolution of single millisecond (and even less), simple to operate and inexpensive compared to other devices, EEG still had a number of limitations. By applying EEG methods, brain responses of the patients were recorded with or without visual indications, which bring difficulty for both patients and their caregivers, especially in the later stages of the disease. Large areas of the cortex have to be activated synchronously for ensuring that adequate potentials are generated and changes to be enumerated at the electrodes positioned on the scalp. In addition, the position of the source of the electrical activity may sometimes give puzzling impressions due to the propagation of electrical activity along the physiological pathway or through volume conduction in extracellular spaces. The placement of an EEG cap may also bring discomfort to the patients without making any head movements and this will be a tedious procedure during the data collection [106,107,108]. 

For EMG, this technique is related to the function of muscles through measures of the electrical activity (action potentials) activated during muscular contractions. One of the informative diagnostic EMG signal approach used to measure PD patient’s muscles is through surface (interference) electrode placed on the skin. This signal is frequently examined using amplitude and spectral analysis techniques. These approaches are applied mainly to calculate the degree of muscle activation and fatigue [109,110,111]. However, the physician that uses the EMG electrodes is requested of having knowledgeable perception on the anatomy of the human body as it is essential for the accurate electrode location and placement. The physician must also ensures that the inter-electrodes distance are constant during the whole experiments for making sure the electrodes are over the identical muscle fibers. Moreover, there are many undesirable signals obtained together with the useful signals, for instance skin artifacts, power line artifacts, motion artifact due to electrodes not attached properly at the skin interface or loose tips of the wires, involuntary reflex activity, and any other electrical device that may be available in the room when data are collected. Besides that, this technique cannot function accurately if the patients had taken medicine beforehand, which will disturb the nervous system, for instance, a muscle relaxant or anticholinergic (medicine that function for reducing uncontrollable movements, relaxing the lung airways, and relieving cramps) [109,110,111,112]. 

While for methods using imaging modalities such as MRI, it also brings some drawbacks where the MRI machines will make a tremendous amount of noise during the operation of the machine. The simultaneous actions of being put in an enclosed space and the loud noises from the machine made by the magnets can cause some patients having a claustrophobic feeling while undergoing the MRI scan. It also requires subjects to maintain still for some period of time, but the MRI scan can take up to 90 min to complete the whole test. A very minor movement during the scan may bring the effects of distorted images meaning that the scanning will require to be taken again. In addition, if multimodal MRI is applied, this modality has additional drawbacks, which include the increased cost of the scan, increased scan time, increased post-processing and reading time, and the need for an experienced radiologist who is familiar with the post-processing and interpretation of images and metabolic spectra produced by these modalities. On the other hand, CT scans have the gains of more precise, painless and more detailed compared to other imaging modalities. However, they insert a high dose of radiation in the patients and sometimes will give misconceptions to physicians where the scan can cause negative effects to the patients’ body if found out that there is a mistake. Then the patients will have to experience unnecessary cures, which exposed them to more radiation [113,114]. 

In latest years, the significance of biomedical engineering and wearable technology for healthcare is developing with the progress and the accessibility of many strategies and technological explanations. With the latest on-going advance development in various technologies and systems, this latest knowledge will permit the monitoring of PD with the application of wearable and user-friendly technology. Recently, wearable sensors (accelerometer, gyroscope and magnetometer) and audio sensor have been taken into consideration to progress the experience and capabilities of doctors and medical specialist in making judgments about the PWP. There is no hesitation that the assessment of data tool from PWP and judgments of experts is still the most significant factors in diagnosis. However, these computational tools and techniques have the potentials of being useful supportive tools for the experts. The developed system can be an assistance in improving the precision and consistency of diagnoses and reducing potential errors, and at the same time making the diagnoses more time saving [115]. Current technological developments in the multimodal miniature sensor system (combination of more than two sensors), which includes mobile and ubiquitous monitoring have been producing excessive growing attention in applying wearable technology for health monitoring. Wearable sensors or body fixed sensors placed on the body to monitor the kinematic and physiological parameters have been advanced to the state that they can be equipped for clinical applications and started to play an important role in patient’s daily routine. The success of these wearable sensor technology fully depends on the sensor performance, cost and reliability. For these reasons, wearable sensors have become very useful for scientific applications and in daily life settings-home monitoring. The use of wearable sensors for monitoring at home has the prospective to expand the quality of delivering healthcare while creating it to be proficient in the process of rehabilitation. This allows physicians resolving restrictions of ambulatory technology and providing feedback for physicians in order to monitor individuals over weeks or even months [115,116]. 

The main target using this wearable technology is providing an objective evaluation of motor disorder status, for instance, PD through the motion analysis. Most recently, body-fixed sensors such as accelerometers, gyroscopes and magnetometers have been widely used for PWP mobility monitoring, especially in term of recording their daily activities. Perhaps, the researchers begin exploring PD motor disorders and the likelihood of employing wearable technology for assessing the effect of clinical interventions on the value of movement observed while PWP accomplished tasks required. These sensors have turn out to be smaller, more robust, totally unobtrusive and precise in the previous couples of years back that facilitate long-term monitoring [117,118,119]. An accelerometer is a low-cost, flexible and miniature devices that provide sufficient information for human motion detection in clinical/laboratory settings or free-living environments. This sensor has been the most commonly used wearable sensor in the field of physical activity recognition and monitoring. It is a type of position sensor functioned by measuring acceleration in motion along each reference axis. Measuring human physical activity using accelerometer is preferred because acceleration is proportional to external forces and therefore reflects the intensity and frequency of human movement. A gyroscope measures angular rotation of body segments, when attached to the segment with their axis parallel to the segment axis. It uses the vibrating mechanical element to sense angular velocity (angular rate) along one rotational axis. It can measure transitions between postures by measuring the Coriolis acceleration from rotational angular velocity and often combined with accelerometers in human motion studies. Magnetometer measures a change in rotation of the body segment with respect to the earth’s magnetic field. The general concepts of these sensors correspond to the magneto-resistive effect, which is the property to change the resistance with a change in magnetic induction. Magnetometer is mostly combined with inertial sensors (gyroscope and accelerometer) where every sensor has their own benefits for overall recognition performance. The combination of multimodal sensors (accelerometer, gyroscope and magnetometer) forms an inertial measurement unit (IMU) that provides quick, accurate position and orientation determination with a low amount of drift over time [117,118,119,120,121].

Besides the application of wearable sensors, research also shown that speech signal may be a useful biomarker to remotely monitor PD symptom severity based on the sources of medical indication that suggested the huge majority of PD patients usually reveal some form of vocal disorder. There is strong supported proof of degrading in voice with PD progression. In fact, speech impairment might be among the initial sign of PD symptoms, measurable up to five years prior to clinical diagnosis. Study of progression and severity of PD using speech signals is a non-invasive technique, easy to obtain that drawn significant attention. In addition, speech signals fit ideally the purpose of telemonitoring in medical care, because they can be self-recorded, easy to obtain, potentially reliable, cost-effective screening of PWP and potentially alleviating the burden of frequent, and often inconvenience, visit to the clinic. This also relieves national health systems from excessive additional workload, decreasing the cost and increasing the accuracy of clinical evaluation of the patient’s disease condition [82,83,84,85,86,87,88]. From earlier investigation conducted, there have been a number of initiatives from previous researches addressing the application of wearable sensors that had the ability to enumerate the different types of PD symptoms (*i.e*., dyskinesia, bradykinesia, tremor, FoG, *etc*.) using uni-modal sensor or bi-modal sensors (accelerometer and gyroscope) and application of speech in discriminating healthy control from PWP. 

**Figure 10:**
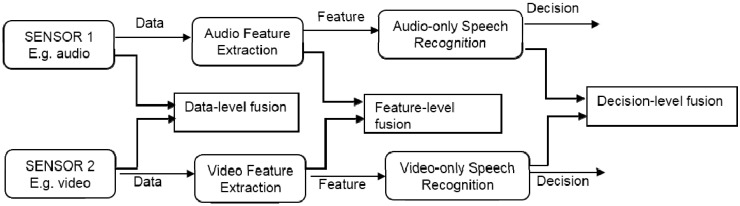
Levels of multimodal fusion (adapted from [124]).

Until now, there is insufficient research on the development of multimodal sensor platform for accurately and efficiently follow PD progression at more frequent intervals with less cost and minimal waste of resources. At the same time, the strength of existing signal processing and classification algorithms was not tested using the information from the combination of multiple sensors. Although many improvements have been shown, but there is still an absence of a multimodal fusion system that had ability to deliver a trustworthy validation of PWP status and at the same time economically practical [116,121]. The driving principle of multimodal fusion (also known as multimodal signal integration) system is computer systems provided with multimodal proficiencies for human/machine interaction and the ability to interpret information from various sensory and communication channels. Multimodal interfaces process two or more combined user input modes, such as speech, gesture, and body movements in a coordinated manner with multimedia system output. Fusion of input modalities is one of the features that distinguish multimodal interfaces from unimodal interfaces. The aim of fusion is to extract useful information from a set of input modalities and pass it to a human-machine dialog manager. Fusion of different modalities is a delicate task, which can be executed at three levels: at data level, at feature level and at decision level. Each fusion scheme operates at a different level of analysis of the same modality channel as illustrated in Figure 10. Data-level fusion is applied for multiple raw data coming from a same type of modality source, for instance, similar scene recorded from two webcams from different viewpoints. The advantage of this fusion is achieving the highest level of information details as the signal is directly processed, but it is highly susceptible to noise and failure as there is an absence of preprocessing. Next, feature-level fusion is a general type of fusion when closely-coupled modalities are to be fused. The typical example is the fusion of speech and lip movements. This level of fusion will produce a moderate level of information details, but less sensitive to noise and failures. Finally, the decision-level fusion is the most common type of fusion in multimodal applications. The key reason is due to its ability to manage loosely-coupled modalities, for instance, pen and speech interaction. This level of fusion is highly resistant to noise and failure as well as improving reliability and accuracy of semantic interpretation, by combining information coming from each input mode [122,123,124].

The rising interest in the design of multimodal sensor fusion platform has been motivated through the benefits of pursuing robustness and providing more convenient, obvious, and powerfully expressive means of human-computer interaction. The multimodal sensor interface design could have potential for more interesting applications, provide access to a larger range of consumers and provide more adverse habit surroundings comparisons to before. These sensor designs regularly reveal improvements when handling errors, reducing recognition uncertainty and demonstrate performance advantages. Perhaps, most importantly, the multimodal sensor system can achieve error suppression higher compared to a unimodal sensor system that improves the overall recognition rates [123,124]. The prospective gain obtained when fusing information from numerous sensors corresponds respectively to the notions of overlapping, complementarily and timeless provided for the system. Overlapping information sources provided from integration of two or more sensors obtained through a multimodal interface can be an effective means of considerably lessen the overall recognition doubt and thus aid to improve the precision whereby the features are perceived by the system. Additionally, this overlapping information can also serve to improve reliability in the case of sensor error or failure. Complementary information obtained through numerous types of sensors will allow features in the surroundings to be perceived, which are impossible to be perceived using information from every single sensor functioning independently. Increasing the quantity of input sensors interpreted within the multimodal system can provide more appropriate information as compared to single sensor due to either the processing parallelism or the speed of each unimodal sensor, which had possibility to achieve as part of the integrating. Overall, a well-designed multimodal sensor interfaces fusing two or more information sources can successfully function in a more robust, reducing the recognition uncertainty and stabilizing the system performance compared to unimodal system that involve only a single recognition technology [124,125,126,127,128,129]. 

The latest studies have raised the significant topic of looking for a statistical mapping between speech properties and application of wearable sensors as an issue worthy of advance exploration. The combination of wearable sensors (accelerometer, gyroscope and magnetometer) and audio sensor can be an appropriate to investigate, on the basis of clinical evidence, suggesting that the earliest prodromal PD symptoms in the vast majority of PWP are slowness (82.4%), difficulty in walking (77.1%), difficulty in writing (53.6%), stiffness (50%), tremor (82%) and speech difficulty (34%) [130]. On one hand, wearable sensor technology is totally unobtrusive and does not interfere with the PWP’s normal behavior. While on the other hand, it has been suggested that speech is affected in the early stage where it is a natural candidate for measuring and quantifying the progress of PD. With the benefits from both wearable sensors and audio sensor as the biomarker of PD assessment, the fusion of these two sensors is expected to deliver an outstanding performance in management related to PD and provide a remarkable improvement in the patients’ management as well as a substantial cutting-off of the economic burden caused by PD. Currently, to the authors’ knowledge, the latest research on multimodal sensor fusion do not cover the focus on a combination of wearable sensors (accelerometer, gyroscope, and magnetometer) and audio sensor for monitoring the progression of PD. For this reason, areas for future research focused on the integration of multimodal sensor fusion with wearable sensors and audio sensor as the biomarker for enriching early diagnosis of PD are proposed. 
